# Serum insulin-like growth factor-1 as a potential prognostic biomarker for heart failure with reduced ejection fraction: a meta-analysis

**DOI:** 10.3389/fcvm.2024.1415238

**Published:** 2024-09-17

**Authors:** Tingting Liu, Fangyu Li, Yihuan Fei, Fangling Sun, Mengqi Chen, Xin Tian, Wenrong Zheng, Zixin Zhu, Wen Wang

**Affiliations:** ^1^ Department of Experimental Animal Laboratory, Xuanwu Hospital of Capital Medical University, Beijing, China; ^2^ Innovation Center for Neurological Disorders and Department of Neurology, National Clinical Research Center for Geriatric Diseases, Xuanwu Hospital of Capital Medical University, Beijing, China; ^3^ School of Chemical and Pharmaceutical Engineering, Hebei University of Science and Technology, Shijiazhuang, Hebei, China; ^4^ Beijing Institute for Brain Disorders, Beijing, China

**Keywords:** heart failure, IGF-1, meta-analysis, prognostic biomarker, HFrEF

## Abstract

**Background:**

Most studies have indicated that peripheral insulin-like growth levels factor-1 (IGF-1) is valuable in diagnosing heart failure, although the results have been inconsistent. To help solve the debate, we performed a meta-analysis to explore the relationship between IGF-1 and heart failure (HF).

**Methods:**

We conducted an extensive search across various databases such as Embase, Cochrane Library, Pubmed, Medline, and Web of Science on May 30, 2023. From the extensive pool of studies, we selected 16 relevant articles, encompassing a total of 1,380 cases and 1,153 controls, to conduct a rigorous meta-analysis.

**Results:**

The total results indicated that there is an association between lower IGF-1 level and HF. The random-effects model yielded a pooled standardized mean difference (SMD) of −0.598 (95% CI: −1.081 to −0.116, *P* = 0.015). Further subgroup analysis also showed that IGF-1 levels were associated with HF in the age difference ≥5 years subgroup and body mass index difference >1 subgroup. Additionally, significant association between IGF-1 levels and HF were detected in the “serum” samples and “Europe” subgroups. Importantly, we observed IGF-1 showed significant lower levels in patients with reduced ejection fraction (HFrEF) compared to the controls, not in patients with preserved ejection fraction (HFpEF). The Begg’s and Egger’s tests revealed no indication of publication bias.

**Conclusions:**

Our meta-analysis has provided evidence suggesting a substantial correlation between reduced levels of IGF-1 and the occurrence of HF. Further prospective studies are necessary to ascertain the use of IGF-1 as a reliable biomarker for diagnosing HF, especially for HFrEF. But the diagnosis of HFpEF should be cautious.

## Introduction

Heart failure (HF), which affects approximately 64.3 million people globally, is the primary cause of death for those afflicted with cardiovascular diseases (1). The global prevalence of heart failure is expected to rise rapidly in the upcoming decades (2). Despite notable advancements in diagnostic and treatment approaches for heart failure in recent years, this condition continues to be a significant contributor to morbidity and mortality globally. Therefore, it is crucial to explore the underlying mechanisms of this condition and develop more efficacious diagnostic and treatment methods.

Identifying HF patients at an early stage who are at a higher risk can result in timely intervention, which has the potential to enhance outcomes (3). In recent decades, there has been an abundance of studies investigating prognostic biomarkers for HF; however, it is still difficult to predict both short-term and long-term outcomes. Several biomarkers, including B-type natriuretic peptide (BNP) (4), N-terminal proBNP (NT-proBNP) (4), sST2 (5), Gal3 (6), and GDF-15 (7) have been previously identified as having prognostic value for HF. Current guidelines recommend only BNP and NT-proBNP among these biomarkers.

The growth hormone/insulin-like growth factor-1 (GH/IGF-1) system is crucial for regulating growth and cellular differentiation in various tissues. IGF-1, homologous to pro-insulin, is a peptide consisting of 70 amino acids and is mainly synthesized in the liver and kidney. It is generally known as an anabolic growth hormone, responsible for cell growth, differentiation, proliferation and survival in almost all body organs, such as the heart, brain, breast, hepatocytes, skin, and so on (8–14). Numerous reports advocate the hypothesis that IGF-1 participates in the homeostasis of cardiovascular physiology. In particular, IGF-1's roles in the cardiovascular system include maintaining cellular homeostasis by regulating vascular vasoconstriction/vasodilatation, cardiac apoptosis and autophagy, and inflammatory responses (15–18). Many studies have attempted to explore the correlation between alterations in serum IGF-1 levels in individuals suffering from HF; nonetheless, researchers have obtained conflicting outcomes. In certain studies, a considerable decrease in serum IGF-1 levels has been observed among HF patients in comparison to control groups (19–25). However, some studies found that HF patients have higher serum IGF-1 levels than normal individuals (26, 27). Therefore, this study aims to conduct a comprehensive and critical meta-analysis of previous studies to arrive at a clearer and evidence-based conclusion regarding the association between serum IGF-1 levels and HF.

This study provided a summary of the research conducted on the correlation between IGF-1 and the risk of heart failure. Initially, our aim is to investigate if there is a connection between heart failure and changes in serum IGF-1 levels. Additionally, we aim to identify potential factors that could explain the significant variations observed in the results reported by these studies.

## Materials and methods

The present systematic review adheres to the Preferred Reporting Items for Systematic Reviews and Meta-Analyses (PRISMA) reporting guideline.

### Search strategy

Two investigators performed an unbiased systematic review of English-language literature using multiple databases included “PubMed”, “Medline”, “Web of Science”, “Cochrane Library”, and “Embase”. The search terms used for each database were “insulin-like growth factor-1 OR IGF-1 AND heart failure.” The search was conducted until May 30, 2023. Additional information and a detailed search record are available in the Supplementary Materials.

### Inclusion and exclusion criteria

The meta-analysis comprised eligible studies meeting the following criteria: (1) exploration of the association between blood IGF-1 concentrations and HF risk; (2) inclusion of a case group of patients with HF and a control group of healthy individuals; (3) utilization of qualified clinical criteria for HF diagnosis; (4) provision of IGF-1 data; (5) studies conducted in human subjects; and (6) availability of full English text.

The study's exclusion criteria were: (1) Insufficient data on IGF-1 concentrations. (2) Sole reporting of a single group of subjects. (3) Repetitive reporting about the same group of subjects. (4) Extraction of heart tissue or postmortem samples. (5) Study types that include non-human studies, case reports, commentaries, reviews, meta-analyses, conference abstracts or unrelated topics.

### Data extraction

Information regarding the first author, publication year, study location (country), study design, number of participants for patients and controls, diagnostic criteria, average age of each group, gender distribution of each group, classification of HF (HFpEF: heart failure with preserved fraction, HFrEF: heart failure with reduced ejection fraction), sample type (serum, plasma), patient medication and detection technology (ELISA: enzyme-linked immunosorbent assay; RIA: radioimmunoassay; IRM: immunochemical assays), BMI, IGF-1 concentrations, the mean and standard deviation (SD) or median and the minimum and maximum values or median and interquartile range (IQR) were recorded from the included studies. If the case or control groups were further segmented into subgroups, the data from each subgroup were combined as $n=n_{1}+n_{2}$, $\bar{x}=\frac{n_{1}{\bar{x}}_{1}+n_{1}{\bar{x}}_{2}}{n_{1}+n_{2}}$ and $\mathrm{SD}=\sqrt{\frac{\left(n_{1}-1){\mathrm{SD}}_{1}^{2}+(n_{2}-1){\mathrm{SD}}_{2}^{2}+\frac{n_{1}n_{2}}{n_{1}+n_{2}}({\bar{x}}_{1}^{2}+{\bar{x}}_{2}^{2}-2{\bar{x}}_{1}{\bar{x}}_{2}\right)}{n_{1}+n_{2}-1}}$.

In instances where a study solely provided medians and IQRs in lieu of means and SDs, and omitted the minimum or maximum values, we regarded the medians as synonymous with to the means. Subsequently, we derived the SDs by dividing the IQR by 1.35 (28). Alternatively, if the study supplied the minimum and maximum values, we estimated the means and SDs, as per the methodology outlined by Hozo et al. (29).

Two investigators carried out independent data extraction to identify any inaccuracies or omissions. Any discrepancies were resolved through discussions including a third author during the extraction process.

### Quality evaluation

The quality of the studies included was appraised through the Newcastle-Ottawa Scale (NOS), suggested by the Agency for Healthcare Research and Quality in the United States. For the purpose of evaluating comparability of design or analysis between cases and controls, age and gender matching was exercised. Studies that secured a score below 5 on the NOS scale were excluded following assessments of low quality (Supplementary Material Table 1).

### Statistical analysis

The relationship between the blood IGF-1 level and HF risk was evaluated by calculating the pooled standardized mean difference (SMD) and the two-sided 95% confidence intervals (CIs) for the meta-analysis. SMD denotes the mean difference in outcomes between cases and controls divided by the pooled standard deviation (SD), and yields a unit-free measure of effect size. The significance of the pooled SMD was determined utilising a *Z* test with the significance level set at *p* < 0.05. Heterogeneity amongst the studies was assessed using the *Q* test and the *I*^2^ statistic. When significant heterogeneity was identified (*p* < 0.1 in the *Q* test, and *I*^2^ > 50%), a random effects model was employed to combine the data from the primary studies. STATA 17.0 software (Stata Corporation, College Station, TX, United States) was used to do the meta-analysis and draw the forest plots.

To examine the potential impacts of age difference, sample type, and study location, we performed the subgroup analysis. Subgroup analyses were carried out based on age difference (<5 years or ≥5 years), BMI difference (≤1 or >1), sample type (serum or plasma) and region (Europe, East Asia, etc.). Random effects models were also used in subgroup analyses through STATA 17.0 software (Stata Corporation, College Station, TX, United States).

Network meta-analysis is a broadened form of pairwise meta-analysis that compares multiple different classifications across several diseases for the same condition (30). We then used the network meta-analysis to compare the IGF-1 value between HFrEF and HFpEF. A test for global inconsistency was performed, using the “network meta i” command, with a *p*-value more than 0.05; therefore, a consistency model that can be fitted was used to perform network meta-analysis. Between-studies heterogeneity was tested using the “network sidesplit” command through STATA 17.0 software (Stata Corporation, College Station, TX, United States). Direct and indirect comparison effects were jointly estimated using the node-splitting method.

A meta-regression using restricted maximum likelihood estimation (REML) was conducted to evaluate the potentially significant covariates exerting a substantial impact on the between-study heterogeneity through STATA 17.0 software (Stata Corporation, College Station, TX, United States). The meta-regression analysis included age difference (<5 years or ≥5 years), sampling type (plasma or serum), and study location (Europe, East Asia, and North America).

To assess result stability, the sensitivity analysis excluded individual studies on a step-by-step basis. Publication bias was evaluated by Begg-Kendall test with *p* < 0.05 representing significant publication bias and displayed using a funnel plot. STATA 17.0 software (Stata Corporation, College Station, TX, United States) was used to carry out all the analyses including meta-analysis, subgroup analysis, meta-regression, and publication bias assessment. A two-sided *p*-value lower than 0.05 was applied as the threshold for significance in all statistical tests.

## Results

### Study selection and characteristics

**Figure 1:**
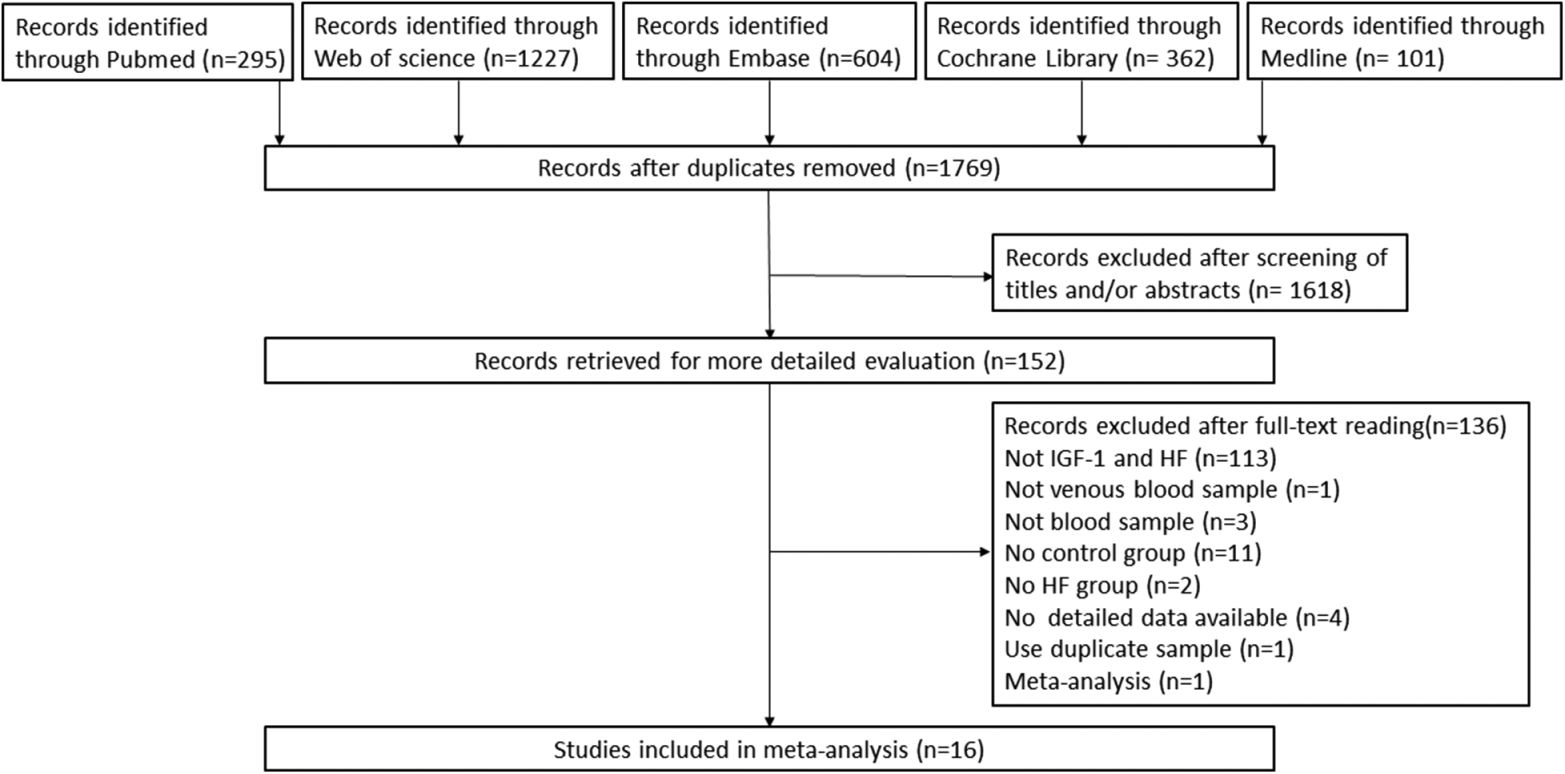
Preferred reporting items for systematic reviews and meta-analyses (PRISMA) flow diagram of the study selection process. HF, heart failure; IGF-1, insulin-like growth factor-1.

The initial search yielded 295 records from Pubmed, 1,227 from Web of Science, 604 from Embase, 362 from the Cochrane Library and 101 from Medline. Following the removal of duplicates and a review of titles and abstracts, a total of 151 pertinent articles on the subject at hand were selected for full-text evaluation. Of these, 136 records were excluded: these were not IGF-1 and HF (*n* = 113), not heart failure (*n* = 1), not blood sample or venous blood sample (*n* = 4), review or meta-analysis (*n* = 1), the reports of studies that was no HF group (*n* = 2), or the studies of which did not have control group (*n* = 11), use duplicate samples (*n* = 1), not detailed data available (*n* = 4). Finally, we identified 16 studies using our inclusion and exclusion criteria. Please refer to Figure 1 for a flowchart showing the process of selecting studies.

Data from the 16 studies used in this meta-analysis were obtained either from the publications or directly provided by the authors where necessary. The characteristics of the studies were described in Table 1 and Supplementary Material Table 2. The total sample sizes consisted of 2,533 participants, comprising of 1,380 cases and 1,153 controls. The average ages of the participants in each study varied from 51.2 to 75.49 years for HF patients and 40.5 to 80 years for HC. Ten studies (62.5%) reported serum IGF-1 levels, and only 37.5% of studies reported plasma IGF-1 levels.

**Table 1:** The detailed characteristics of all the eligible studies for the association with the insulin-like growth factor-1 (IGF-1) levels and heart failure (HF).

Study		Sample size		Mean age		Gender (Male)		Mean IGF-1(±SD) concentrations			Diagnostic criteria
First author	year	HF	HC	HF	HC	HF	HC	HF	HC	unit	
Anker, S. D.	2001	72	26	61	56			142 ± 50.91	151 ± 45.89	ng/ml	NYHA
Jankowska, E. A.	2006	208	366	63	51	208	366	183.26 ± 47.35	298.91 ± 74.48	ng/ml	NYHA
Anwar, A.	2002	103	60	75.49	80	53	27	83.2 ± 49.73	94.6 ± 41.83	ng/ml	NYHA
Broglio, F.	1999	39	42	55.3	56	36	38	135.2 ± 46.8	193.7 ± 63.7	μg/L	NYHA
Saeki, H.	2002	18	15	65.1	56.6	12	10	101.2 ± 34.5	172.9 ± 27.3	ng/ml	NYHA
Faxen, U. L.	2017	164	136	68.34	58	105	68	160.56 ± 71.45	163 ± 53.33	μg/L	NYHA
Kontoleon, P. E	2003	23	20	51.2	40.5	23	20	123.7 ± 50	236.3 ± 66.4	ng/ml	NYHA
Watanabe, S.	2010	142	63	67.8	67.5	103	45	62.2 ± 39.11	42.9 ± 9.48	ng/ml	NYHA
Hu, Z.	2022	95	95	61.49	60.74	51	53	118.25 ± 18.76	165.85 ± 20.47	μg/L	NYHA
D'Assante, R.	2021	13	9	69.64	66.8	10	4	121.38 ± 30.71	160 ± 13.2	ng/ml	NYHA
Andreassen, M.	2009	194	169	69.3	67.2	139	119	78 ± 24.44	77 ± 27.41	ng/ml	NYHA
Al-Obaidi, M. K.	2001	24	21	67	71	17	9	20.2 ± 9.80	14.1 ± 9.17	mU/L	NYHA
Hambrecht, R.	2002	47	15	58	58.5	47	15	175 ± 68.56	170 ± 46.48	ng/ml	NYHA
Barroso, M. C.	2016	77	55	73	54	31	29	99.5 ± 38.67	120 ± 32	ng/ml	LVDD
Guo, S.H.	2022	151	50	71	66	90	21	50.9 ± 26.07	50.1 ± 24.07	ng/ml	NYHA
Toth, M. J.	2006	10	11	63	70	10	11	152.78 ± 63.41	115 ± 29.85	ng/ml	NYHA

HF, heart failure; IGF-1, insulin-like growth factor-1; HC, healthy control; SD, standard deviation; NYHA, New York Heart Association.

### Association between the IGF-1 levels and HF in all eligible comparisons

**Figure 2:**
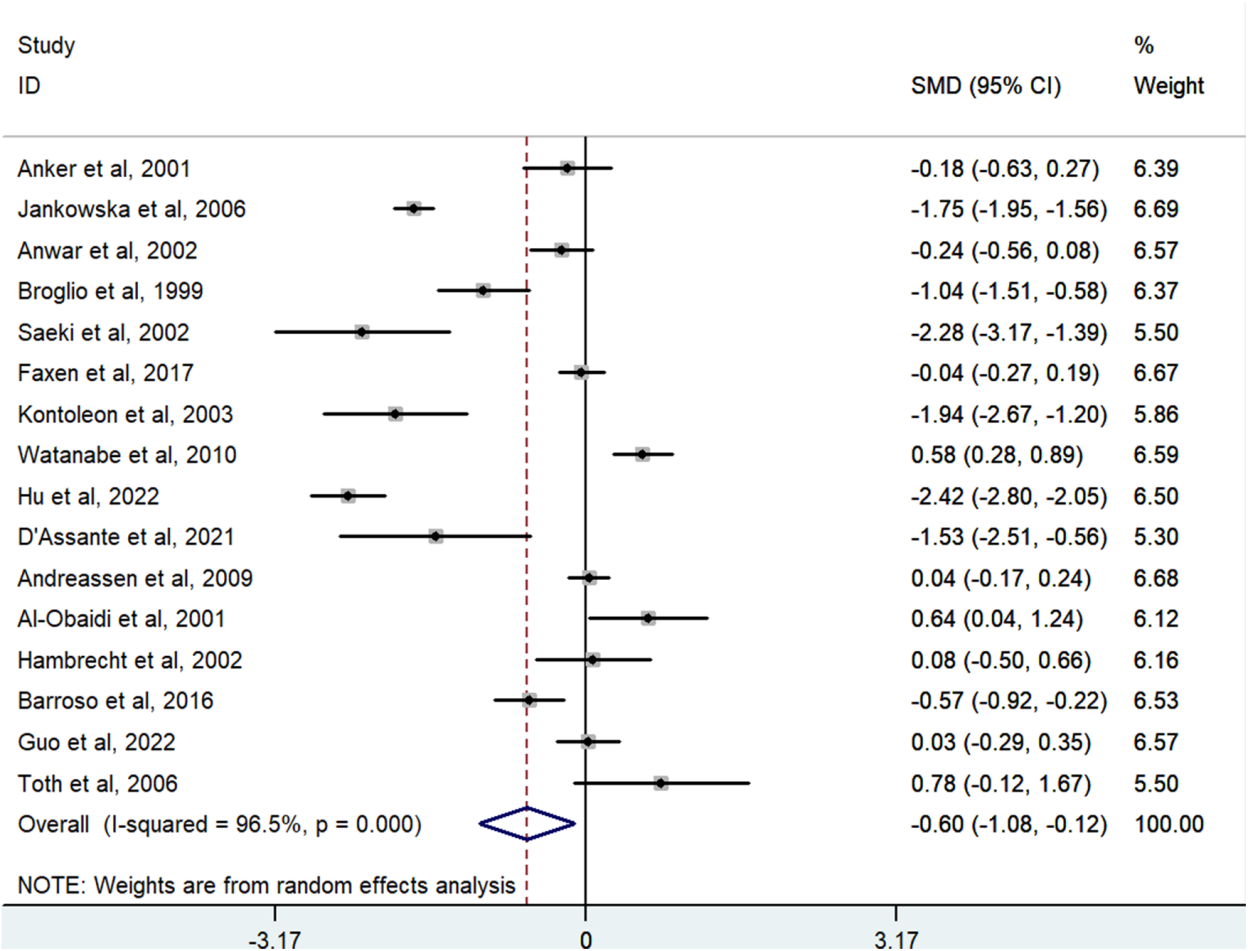
Meta-analysis for the association of the IGF-1 level with HF risk using a random effects model for all included studies. HF, heart failure; IGF-1, insulin-like growth factor-1; SMD, standardized mean difference.

The meta-analysis investigated the correlation between IGF-1 levels and the risk of HF, using data from 16 studies with 1,380 cases and 1,153 controls. The analysis indicated that lower levels of IGF-1 were associated with a higher risk of HF. A random-effects model showed the pooled SMD to be −0.598 (95% CI: −1.081 to −0.116; *P* = 0.015; *I*^2^ = 96.5%) (Figure 2).

### Subgroup analysis

**Figure 3:**
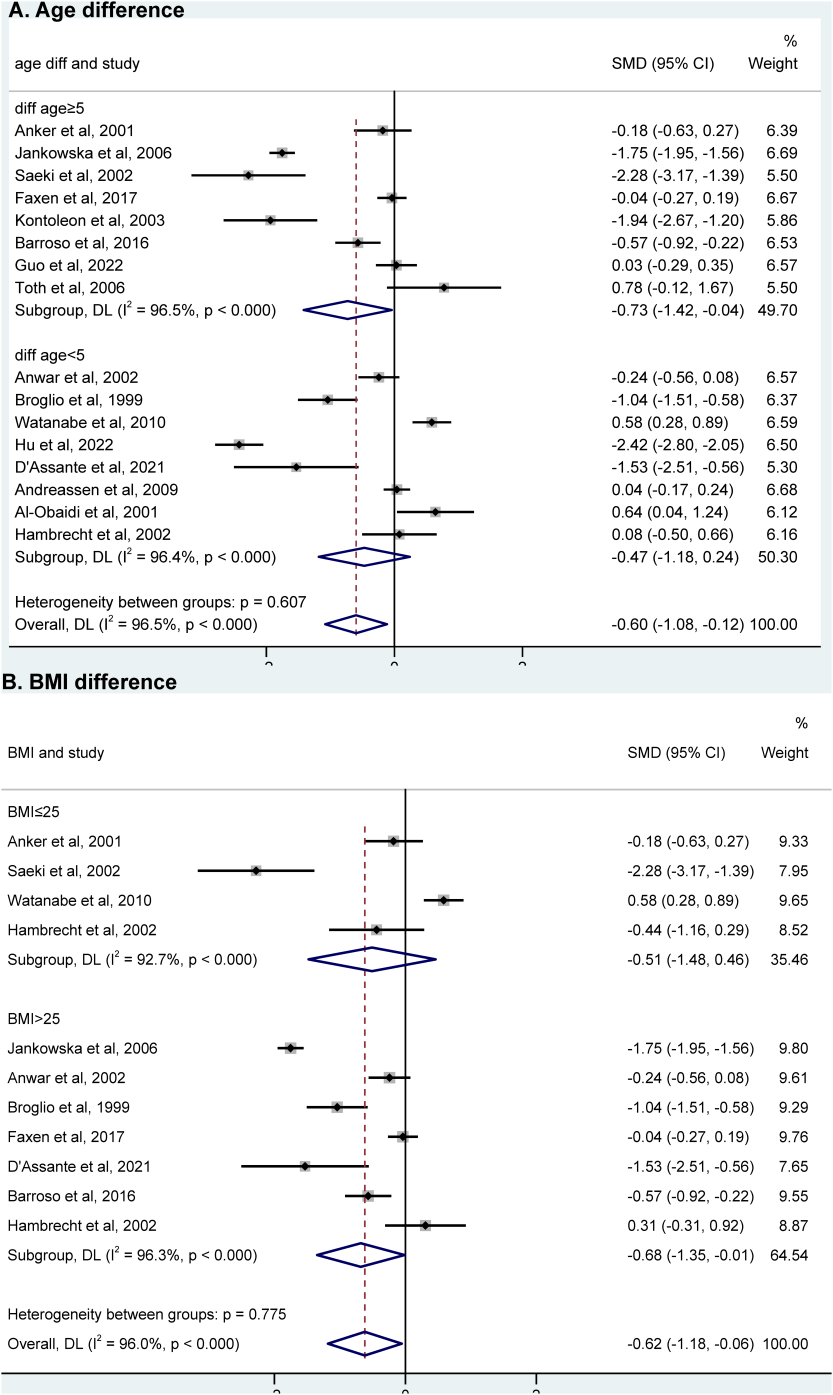
Subgroup analysis of “age difference” **(A)** and “BMI difference” **(B)** using a random effects model between AD group and control group. HF, heart failure; SMD, standardized mean difference; BMI, body mass index.

Among the 16 studies, eight had an age difference greater than or equal to 5 years, while eight had an age difference less than 5 years. When analyzing the subgroups based on age difference, we found that IGF-1 levels were significantly associated with HF in the subgroup of “age difference ≥5 years” [SMD = −0.73, 95% CI (−1.42, −0.04), *P* = 0.038; *I*^2^ = 96.5%], but not in the subgroup of “age difference < 5 years” (Figure 3A). Among the meta-analysis, nine studies included BMI data for HF. These studies were divided into two groups according to the BMI difference between HF and controls: diff BMI ≤1 (four studies) and diff BMI >1 (five studies). IGF-1 levels showed an association with HF in the subgroup of “diff BMI >1” [SMD = −0.75, 95% CI (−1.28, −0.22), *P* = 0.344; *I*^2^ = 87.7%]. However, there was no association observed in the subgroup of “diff BMI ≤ 1” [SMD = −0.60, 95% CI (−1.84, 0.64), *P* = 0.304; *I*^2^ = 98.3%] (Figure 3B).

**Figure 4:**
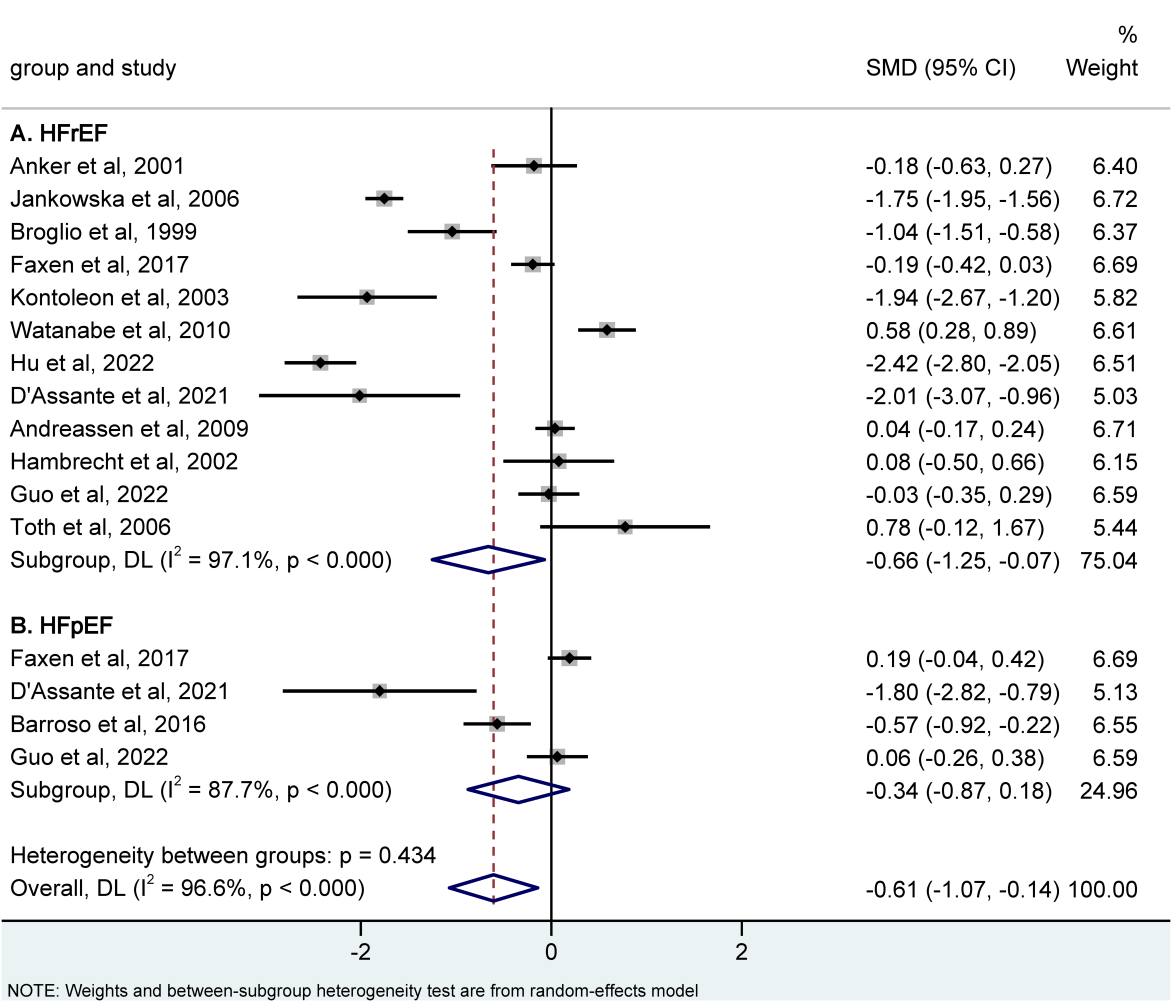
Meta-analysis for the association of the IGF-1 level with hFrEF **(A)** and hFpEF **(B)** risk using a random effects model. HFrEF, heart failure with reduced ejection fraction; HFpEF, heart failure with preserved ejection fraction; IGF-1, insulin-like growth factor-1; SMD, standardized mean difference.

Additionally, there were 12 studies that compared HFrEF and controls, and the result revealed that a reduced IGF-1 level was linked to a higher risk of HFrEF. [SMD = −0.66, 95% CI (−1.25, −0.07), *P* = 0.028; *I*^2^ = 97.1%] (Figure 4A). Regarding HFpEF data, only four studies were available, and further analysis of subgroups did not reveal any correlation between IGF-1 levels and HFpEF [SMD = −0.34, 95% CI (−0.87, 0.18), *P* = 0.202; I^2^ = 87.7%] (Figure 4B).

Out of the 16 comparisons, six were samples taken from plasma, while ten were from serum. Analyzing the subgroup of “plasma” revealed no association between IGF-1 and HF [SMD = 0.02, 95% CI (−0.28, 0.33), *P* = 0.890; *I*^2^ = 72%]. However, subgroup analysis of “serum” showed a significant association between IGF-1 and HF [SMD = −1.03, 95% CI (−1.73, −0.33), *P* = 0.004; *I*^2^ = 97.3%] (Supplementary Material Figure 1). Furthermore, we conducted subgroup analyses based on region, dividing the studies into Europe, East Asia, and North America. The results indicated that only the subgroup analysis of “Europe” showed a significant association between IGF-1 and HF [SMD = −0.57, 95% CI (−1.08, −0.06), *P* = 0.029; *I*^2^ = 95.8%]. IGF-1 levels were not found to be associated with HF in the subgroup analyses of other regions (Supplementary Material Figure 2).

### Compare the IGF-1 value between HFrEF and HFpEF

To discriminate between HFrEF and HFpEF, we assessed the value of IGF-1 in these groups using network meta-analysis (Supplementary Material Figure 3). Comparisons with controls, IGF-1 showed a significant lower level in HFrEF [SMD = −0.7, 95% CI (−1.25, −0.15)] not in HFpEF [SMD = −0.23, 95% CI (−1.12, 0.65)]. When comparing HFrEF and HFpEF, no significant difference was observed [SMD =0.46, 95% CI (−0.45, 1.38)].

### Meta-regression analyses

Meta-regression analysis was carried out in order to explore the influence of sample source, age difference, and region on the effect size of the study. The outcomes uncovered that the sample source can account for 31.54% of the variation between studies (coefficient = −1.13, SE = 0.46, *t* = −2.49, *P* = 0.028) (Supplementary Material Table 3).

### Publication bias and sensitivity analysis

The Begg's and Egger's tests revealed no indication of publication bias (*P* = 0.386, *P* = 0.260, respectively). Additionally, the funnel plot for the overall results also did not exhibit any noteworthy bias (Supplementary Material Figure 4).

During the sensitivity analysis, we evaluated the impact of each eligible study on the combined SMD by excluding one dataset at a time. We observed no change in the corresponding pooled SMD or the statistical significance of the results (Supplementary Material Figure 5), indicating that our findings were highly resilient to the study-selection process.

## Discussion

Our meta-analysis included 16 reports, comprising a total of 1,380 cases and 1,153 controls. The findings showed a significant decrease in serum IGF-1 levels in HF patients compared to the control group. Subgroup analysis was conducted on various factors, including sample type (serum, plasma), geographic locations (Europe, East Asia, and North America), age difference (≥5, <5), BMI difference (≤1, >1), and LVEF (HFpEF ≥ 50%, HFrEF < 50%). We discovered that larger age differences and BMI difference between the case and control groups led to greater differences in IGF-1 levels between the groups. Additionally, subgroup analysis demonstrated a significant association between IGF-1 levels and HF in the “serum” samples and “Europe” subgroups. Importantly, significant differences in IGF-1 levels between HF and controls were observed in HFrEF subgroup not in HFpEF subgroup.

The GH/IGF-1 axis is considered the most potent natural anabolic system, driving post-natal growth mainly by promoting the elongation of bones and the development of muscle mass (31). IGF-1 is a pleiotropic factor, responsible for cell growth, differentiation, proliferation and survival. Despite systemic effects, IGF-1 exerts a wide array of influences in the cardiovascular system affecting metabolic homeostasis, autophagy, hypertension, cardiac contractility and hypertrophy, cardiac fibrosis, and inflammatory (32). It has been shown that IGF-1 prevents starvation-induced cardiac autophagy by increasing intracellular ATP levels, mitochondrial metabolism, mitochondrial Ca^2+^ uptake, and oxygen consumption (33). Norling et al. reported that IGF-1 deficiencies induce pathological vessel wall remodeling that result in a weakening of the cerebral arteries and are associated with functional maladaptation to hypertension (34). In addition, some pieces of evidence are suggesting IGF-1 protects against angiotensin II-induced cardiac fibrosis by targeting αSMA (35). Furthermore, it was found that IGF-1 attenuates the pro-inflammatory phenotype of neutrophils in myocardial infarction (18). The mechanisms explaining the connection between serum IGF-1 levels and HF remain incompletely comprehended. In patients with HF, the hemodynamic impairment is likely to change the blood flow in the pituitary gland, primarily due to the stagnation of venous drainage and/or poor supply of arterial blood. This alteration can result in the death of cells and subsequently cause a deficiency in IGF-1 (36). Besides, heart failure patients often suffer from secondary pulmonary hypertension and subsequently develop right heart failure and backward liver congestion, which may further hinder the secretion of IGF-1 (37). Isgaard et al. has been reported that IGF-1 can increase the contractility of cardiomyocytes primarily through elevating the level of intracellular calcium and sensitizing the myofilaments to calcium, and it can preserve capillary density. Therefore, decreased levels of IGF-1 can also exacerbate the process of HF (32). Research has shown that levels of serum IGF-1 were decreased in heart failure patients, and lower levels of peripheral IGF-1 were linked to an increased risk of heart failure (38). Our research also confirmed this. After exercise training treatment for HF, the lower IGF-1 level was also relieved with the improvement of cardiac function (39). These studies suggest that IGF-1 has the potential to be a disease biomarker in patients with HF.

Studies have recorded that HFrEF and HFpEF may exhibit different behavior regarding the anabolic drive, with HFrEF being altered and HFpEF remaining unmodified (40, 41). In this context, circulating protein biomarkers may offer valuable insights into early physiological changes and biological pathways that contribute to the subsequent development of HF, with the potential for improved understanding of similarities and differences in disease pathogenesis for HFrEF and HFpEF. Regarding the GH/IGF-1 axis, we found that HFrEF patients show a significant decrease in IGF-1 expression compared to healthy controls, and HFpEF patients do not. This finding may be related to the various regulatory roles of IGF-1 in the cardiovascular system as mentioned above. Then, we performed a further network analysis, and the results also indicate significance between HFrEF and the control group. This result indicates that IGF-1 is likely to become a specific biomarker for HFrEF. However, more research is needed to validate our findings and to further delineate the specific etiologies of and factors driving HF subtype development due to limited studies with data on this specific association in HF patients.

It was reported that serum IGF-1 peaks during puberty and declines thereafter during aging in humans (42). In our meta-analysis, we found a positive correlation between IGF-1 levels and the risk of HF in the overall analysis. Nevertheless, when we specifically considered studies with an age gap of less than 5 years between HF patients and controls, there was no evidence of a connection between IGF-1 levels and the risk of HF. Furthermore, when we combined the results of the subgroups with an age difference of ≥5 years, we observed a positive relationship between the age difference and the variance in IGF-1 levels among the control and HF groups. Because obesity and increased adiposity can affect GH secretions, it is reasonable to expect an association between BMI and IGF-1 levels, despite controversial results reported for the relationship between the two factors (43, 44). Additionally, several studies have indicated that obesity can result in a decrease in GH secretion, leading to low-normal IGF-1 levels (45–47). On this account, we analyzed the subgroups based on BMI difference. Our results indicated that IGF-1 levels showed an association with HF in the subgroup of “diff BMI >1”. However, there was no association observed in the subgroup of “diff BMI ≤ 1”. Thus, it is possible that age and BMI are two confounding factors, and the disparity in serum IGF-1 levels between the HF and control groups may be influenced by age difference and BMI difference. Hence, it is highly recommended that future studies incorporate age matching and BMI matching as two inclusion criterions for both groups, and meticulously consider the age and BMI of the control group.

In this meta-analysis, we observed that the lower IGF-1 level is related to HF risk. Our sensitivity analysis demonstrated that the variation caused by any individual study was not significant. Furthermore, we determined the absence of publication bias through the visualization of a funnel plot and the implementation of Begg's test. However, it is important to note that our study does possess certain limitations. Firstly, the number of studies included in our analysis is limited, and therefore, further research may be necessary to strengthen and support our findings. Secondly, there was a statistically significant heterogeneity among the trials, suggesting that caution should be exercised when drawing any conclusions. Additionally, we did not include conference proceedings, potentially resulting in the omission of some minor unpublished studies. Consequently, a detection bias cannot be completely ruled out in our analysis.

## Conclusions

In conclusion, the evidence available supports an association between IGF-1 levels and HF. These findings indicate that serum IGF-1 has the potential to evaluate individuals with HF, especially for HFrEF. Its implementation can improve the identification of individuals with heart failure, offering an opportunity for early diagnosis in clinical practice. But caution is needed when diagnosing HFpEF.

## Data Availability

The original contributions presented in the study are included in the article/Supplementary Material, further inquiries can be directed to the corresponding author.
