# Supplementary material for: Serum insulin-like growth factor-1 as a potential prognostic biomarker for heart failure with reduced ejection fraction: a meta-analysis
Source: Front Cardiovasc Med. 2024 Sep 17;11:1415238. doi: 10.3389/fcvm.2024.1415238 (PMC11442213; doi:10.3389/fcvm.2024.1415238)
Supplement: Supplementary file 1 [file Datasheet1.pdf]

Supplementary files

Title: Serum Insulin-Like Growth Factor-1 as a Potential Prognostic Biomarker for Heart Failure: A Meta-Analysis

Contents:

Supplementary Figure 1. Sampling source type (plasma or serum) subgroup analysis using a random effects model between HF group and control group.

Supplementary Figure 2. Study location subgroup analysis using a random effects model between HF group and control group.

Supplementary Figure 3. Forest plots of network meta-analysis results.

Supplementary Figure 4. The results of the sensitivity analysis of the IGF-1 level with HF risk.

Supplementary Figure 5. Begg's funnel plot for publication bias test of IGF-1 level with HF risk.

Supplementary Table 1. The quality assessment of the included studies based on scores of Newcastle-Ottawa Scale

Supplementary Table 2. The other detailed characteristics of all the eligible studies for the association with the Insulin-Like Growth Factor-1 (IGF-1) levels and heart failure (HF)

Supplementary Table 3. Meta_regression between sampling source type, age difference, study locate and SMD.

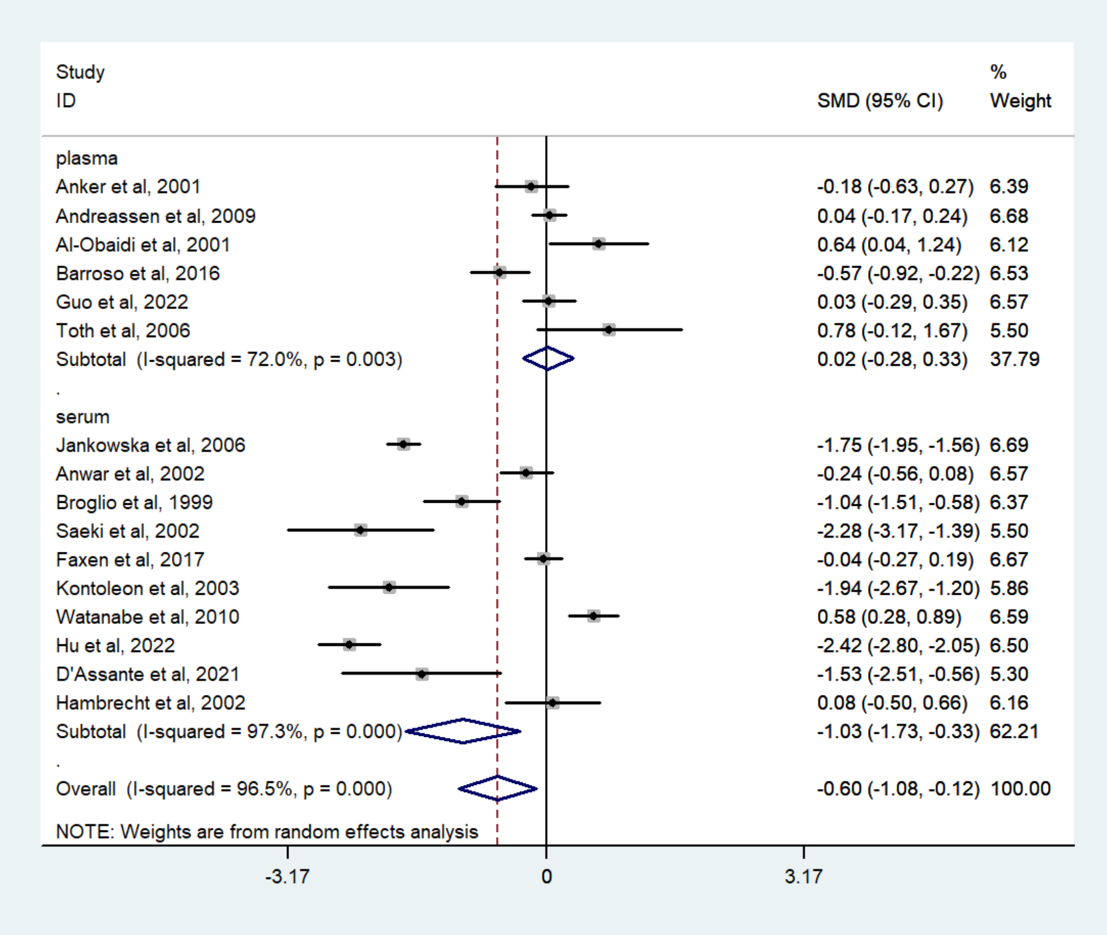

Supplementary Figure 1. Sampling source type (plasma or serum) subgroup analysis using a random effects model between HF group and control group. Note: HF, heart failure; SMD, standardized mean difference;

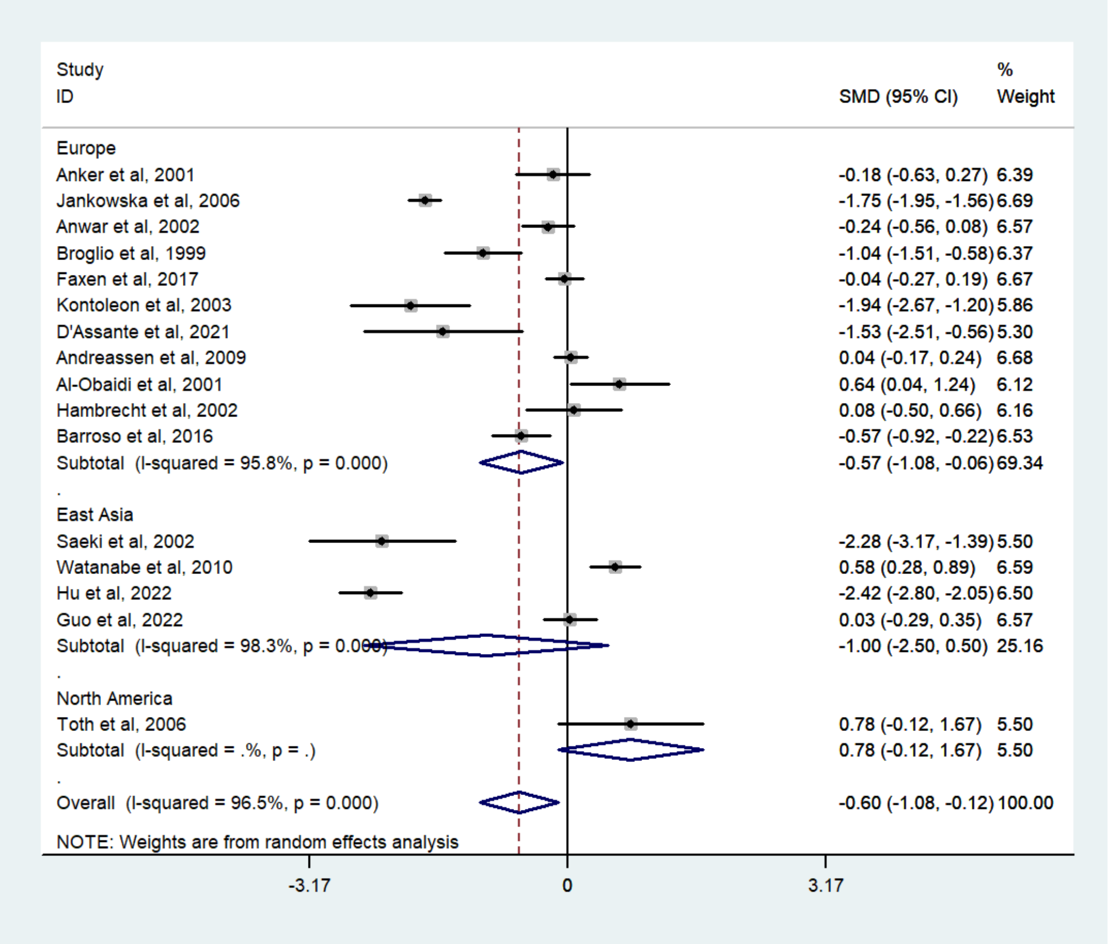

Supplementary Figure 2. Study location subgroup analysis using a random effects model between HF group and control group. Note: HF, heart failure; SMD, standardized mean difference;

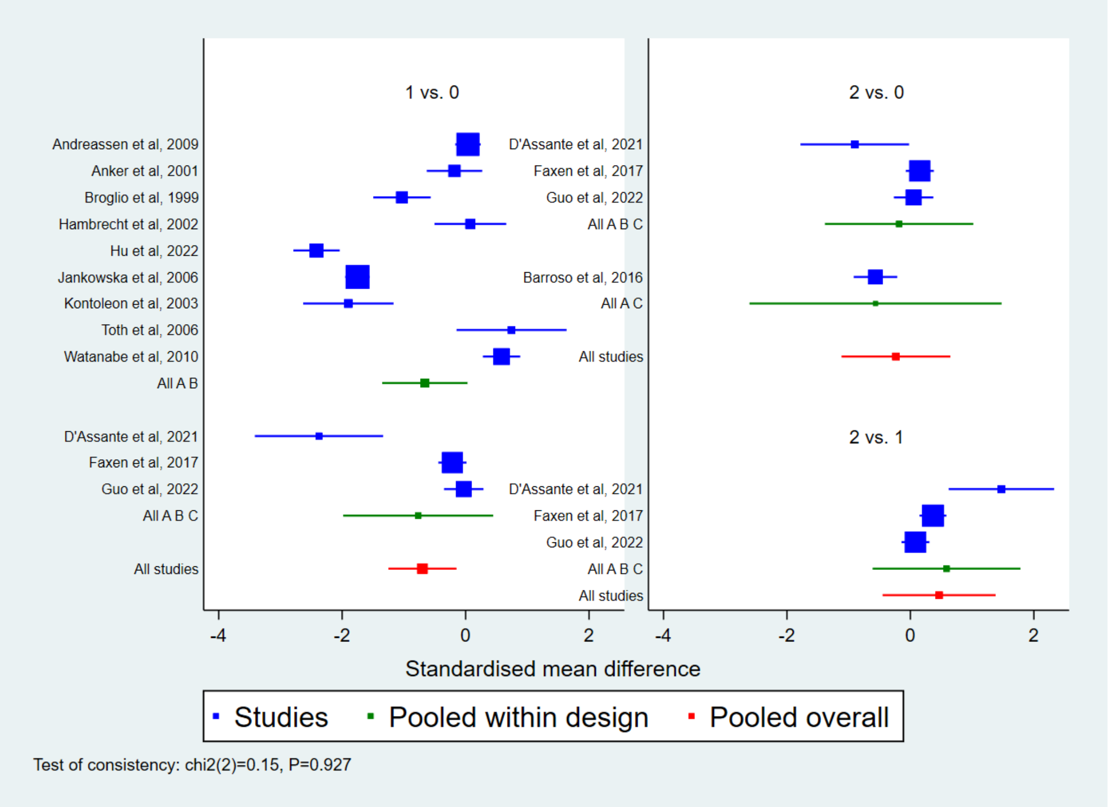

Supplementary Figure 3. Forest plots of network meta-analysis results. Note: 0, controls; 1, HFrEF; 2, HFpEF; HFrEF, heart failure with reduced ejection fraction; HFpEF, heart failure with preserved ejection fraction; SMD, standardized mean difference;

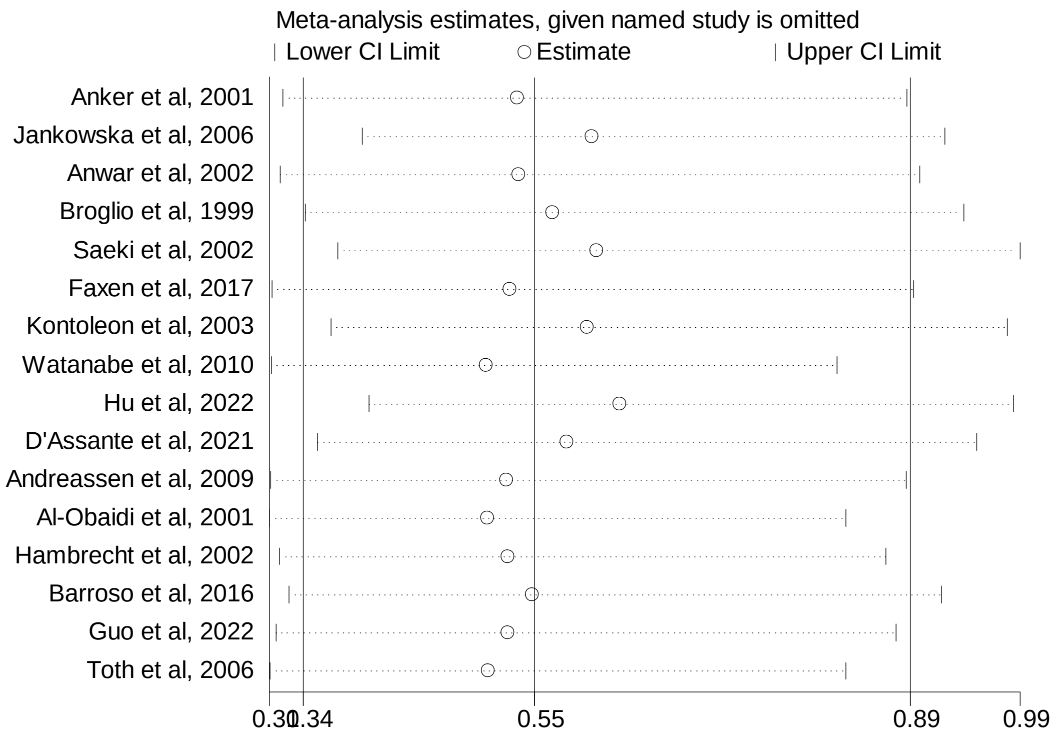

Supplementary Figure 4. The results of the sensitivity analysis of the IGF-1 level with HF risk.

Note: HF, heart failure; IGF-1, Insulin-Like Growth Factor-1;

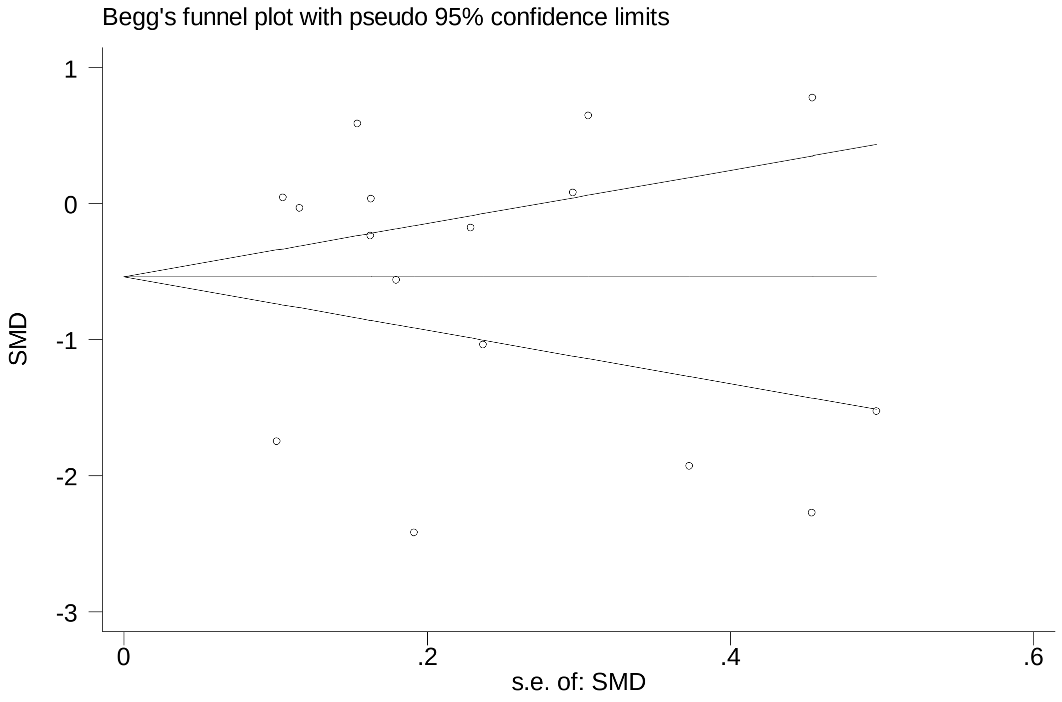

Supplementary Figure 5. Begg's funnel plot for publication bias test of IGF-1 level with HF risk. Note: HF, heart failure; IGF-1, Insulin-Like Growth Factor-1;

Supplementary Table 1. The quality assessment of the included studies based on scores of Newcastle-Ottawa Scale

Author, year	Case definition adequate	Representativeness of the cases	Selection of controls (selected from different diseases or multicenter)	Definition of Controls	Comparability of cases and controls (age and gender)	Ascertainment of exposure	Same method of ascertainment for cases and controls	Non-Response Rate	Total quality scores
Anker et al, 2001	1	1	0	1	1	1	1	NI	6
Jankowska et al, 2006	1	1	0	1	1	1	1	NI	6
Anwar et al, 2002	1	1	0	1	2	1	1	NI	7
Broglio et al, 1999	1	1	0	1	2	1	1	NI	7
Saeki et al, 2002	1	1	0	1	2	1	1	NI	7
Faxen et al, 2017	1	1	1	1	0	1	1	NI	6
Kontoleon et al, 2003	1	1	0	1	2	1	1	NI	7
Watanabe et al, 2010	1	1	0	1	2	1	1	NI	7
Hu et al, 2022	1	1	0	1	2	1	1	NI	7
D'Assante et al, 2021	1	1	0	1	2	1	1	NI	7
Andreassen et al, 2009	1	1	1	1	1	1	1	NI	7
Al-Obaidi et al, 2001	1	1	0	1	2	1	1	NI	7
Hambrecht et al, 2002	1	1	0	1	2	1	1	NI	7
Barroso et al, 2016	1	0	0	1	1	1	1	NI	5
Guo et al, 2022	1	1	0	1	1	1	1	NI	6
Toth et al, 2006	1	1	0	1	2	1	1	NI	7

Supplementary Table 2. The other detailed characteristics of all the eligible studies for the association with the Insulin-Like Growth Factor-1 (IGF-1) levels and heart failure (HF)

Study		BMI		Sample source	measurement method	location
First author	year	HF	HC			
Anker, S. D.	2001	25±4.24	26±5.09	plasma	RIA	Germany Kingdom Italy
Jankowska, E. A.	2006	26.5±4.3	26.9±3.26	serum	immunoassays	Poland
Anwar, A.	2002	25.66±5.97	24±7.75	serum	ELISA	Switzerland
Broglio, F.	1999	25.3±3.2	24.9±1.5	serum	immunoradiometric assay	Italy
Saeki, H.	2002	21.9±3.8	24.5±2.3	serum	ELISA	Japan
Faxen, Ulrika Ljung	2017	27.45±5.56	25±2.96	serum	RIA	Sweden
Kontoleon, P. E	2003			serum	RIA	Greece
Watanabe, S.	2010	21.9+3.8	22.6±2.2	serum	ELISA	Japan
Hu, Z.	2022			serum	RIA	China
D'Assante, R.	2021	25.78 ± 3.1	27.1 ± 5.6	serum		Italy
Andreassen, Mikkel	2009			plasma	ELISA	Denmark
Al-Obaidi, M. K.	2001			plasma	RIA	UK
Hambrecht, R.	2002	22.5±0.5	29.0±0.4	serum	competitive solid phase immunoassay	Germany
Barroso, M. C.	2016	27.5±4.67	25.5±3.7	plasma	ELISA	Germany
Guo, Shaohua	2022			plasma	ELISA	China
Toth, M. J.	2006			plasma	RIA	USA

Note: HF, heart failure; RIA, radioimmunoassay; ELISA, enzyme-linked immunosorbent assay; HC, healthy control; SD, standard deviation; BMI, body mass index.

Supplementary Table 3. Meta_regression between sampling source type, age difference, study locate and SMD.

SMD	Coef.	Std.Err.	t	P	95% CI
sampling source	-1.13	0.46	-2.49	0.028	-2.13- -0.14
study locate	-0.21	0.40	-0.52	0.61	-1.09 – 0.67
age difference	-0.067	0.034	-1.95	0.075	-0.14 - 0.008
cons	1.79	1.04	1.73	0.11	-0.47 – 4.05

Note: SMD, standardized mean difference; Coef., confidence; CI, confidence interval; Std.Err. standard error.
